# Biosurfactants: Potential Agents for Controlling Cellular Communication, Motility, and Antagonism

**DOI:** 10.3389/fmolb.2021.727070

**Published:** 2021-10-11

**Authors:** Jyoti Sharma, Durai Sundar, Preeti Srivastava

**Affiliations:** Department of Biochemical Engineering and Biotechnology, Indian Institute of Technology Delhi, New Delhi, India

**Keywords:** biosurfactant, quorum sensing, motility, bacteria, biofilm, virulence

## Abstract

Biosurfactants are surface-active molecules produced by microorganisms, either on the cell surface or secreted extracellularly. They form a thin film on the surface of microorganisms and help in their detachment or attachment to other cell surfaces. They are involved in regulating the motility of bacteria and quorum sensing. Here, we describe the various types of biosurfactants produced by microorganisms and their role in controlling motility, antagonism, virulence, and cellular communication.

## Introduction

Surfactants are amphipathic molecules that have a hydrophobic (nonpolar tail) and a hydrophilic (polar head) region. They aggregate along the boundary of different phases of liquid such as oil/water or air/water. When the concentration of these molecules increases beyond a threshold, they form micelles, and the concentration above which they form the micelle is called the critical micelle concentration (CMC) (Desai and Banat, 1997). Micelles reduce the surface tension, a property of liquid to resist external forces, between different phases of liquid. Hence, this property of surfactant is used to remove oil from water or soil. Surfactants can be produced chemically or biologically. However, the excess use of chemical surfactants is hazardous to the environment (P. Singh et al., 2019). Biosurfactants are produced by a wide range of microorganisms such as bacteria, fungi, and yeast as secondary metabolites which are either secreted extracellularly or adhered to the cell surfaces. Biosurfactants can be used to replace chemical surfactants, as they are environment-friendly, less toxic, biodegradable in nature, have higher foaming ability, and possess lower CMC values than the chemical ones. These potential advantages make them useful in several applications such as bioremediation, health care, cosmetics, food, and oil industries (Jahan et al., 2020). Apart from being a surface-active agent, biosurfactants aid in cellular communication.

**FIGURE 1:**
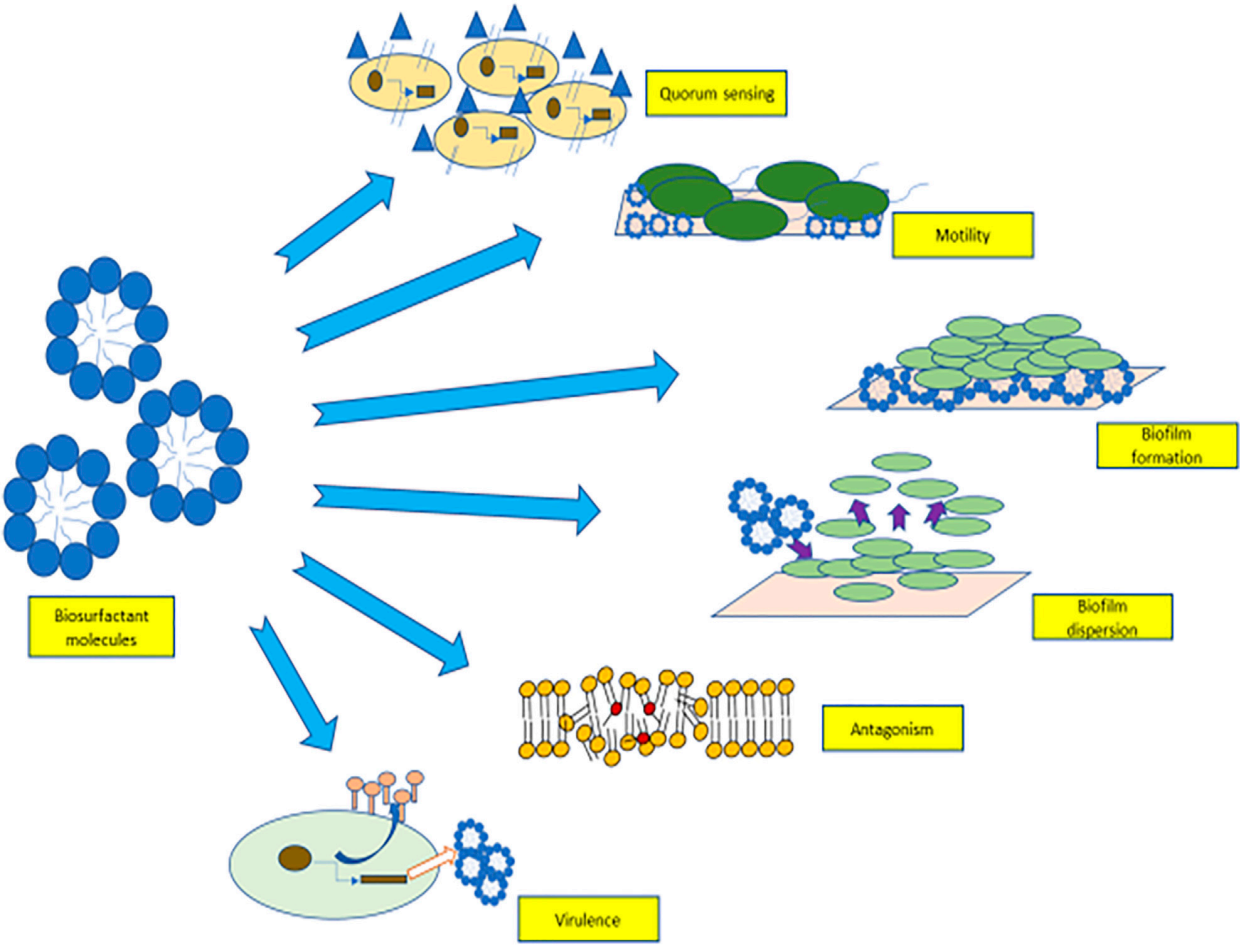
Role of biosurfactants in quorum sensing, motility, biofilm adhesion, biofilm detachement, virulence factors, and antagonism.

Biosurfactants are involved in a myriad of cellular communication methods which are shown in Figure 1. They are used in quorum sensing (ability of certain bacteria to detect and modulate cell population density) (Ibacache-Quiroga et al., 2013), as an antimicrobial agent that participate in microbial competition. Biosurfactant molecules also help in the adhesion and de-adhesion of biofilms from surfaces through cellular communication. These diffusible amphiphilic molecules aid in the survival of microorganisms in the microbial community by binding and sequestering toxic compounds (Gnanamani et al., 2010). Biosurfactants aid in cellular communication, and these features can be used as an alternative approach for their economical production commercially (Banat et al., 2014a).

## Types of Biosurfactants

**FIGURE 2:**
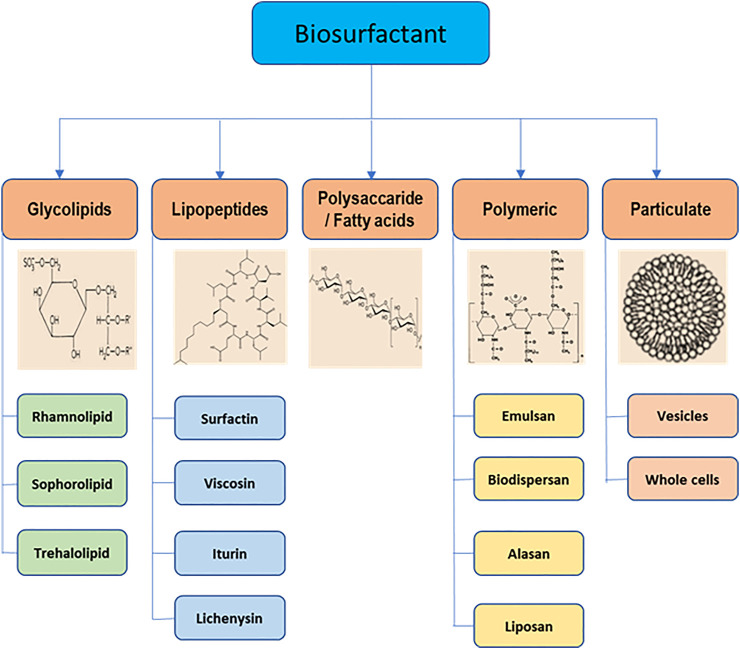
Main classes of biosurfactants and their structures.

Biosurfactants are categorized on the basis of their chemical components (Table 1) (Mulligan and Gibbs, 2004). Generally, the hydrophilic part is an amino acid, peptide, mono-, di-, or polysaccharide, and the hydrophobic part consists of different lengths of fatty acid chains. Biosurfactants are also classified on the basis of their molecular mass. They are divided into low molecular mass compounds such as glycolipids, lipopeptides, proteins, and high molecular mass compounds such as polysaccharides, lipoproteins, and polymeric particles type. Low molecular mass compounds reduce surface tension, whereas high molecular mass compounds are mostly effective in formation of stable emulsions of oil-in water (Rosenberg and Ron, 1999). The important group of biosurfactants and their classes are shown in Figure 2 and described in detail below.

**TABLE 1:** Types of biosurfactant and their sources.

s.no	Class of biosurfactant	Type of biosurfactant	Class of microorganisms	Sources of biosurfactant	References
1	Glycolipids	—	—	—	—
1.1	—	Rhamnolipids	Gammaproteobacteria	*P. aeruginosa, Pseudomonas* sp*. Burkholderia sp*., *T. aquaticus*, and *Meiothermus rubber*	Herman et al. (1997), Hörmann et al. (2010), Maier and Soberón–Chávez (2000), Řezanka et al. (2011)
	—		Gammaproteobacteria		
	—		Betaproteobacteria		
	—		Deinococci		
1.2	—	Sophorolipids	Ascomycetes	*Torulopsis bombicola, T. apicola, Candida kuoi, Rhodotorula bogoriensis,* and *Wickerhamiella domericqiae*	Celligoi et al. (2020), Price et al. (2012), Van Bogaert et al. (2007), Chen et al. (2006), Deshpande and Daniels (1995)
	—		Microbotryomycetes		
	—		Ascomycetes		
	—				
1.3	—	Trehalolipids	Actinobacteria	*Nocardia* sp., *Rhodococcus erythropolis, Mycobacterium* sp*., Corynebacterium* sp.*,* and *Gordonia s*p.	Macdonald et al. (1981), Franzetti et al. (2010)
1.4	—	Mannosylerythritol lipids	Actinobacteria	*Arthrobacter* sp., *Candida antartica, Pseudozyma* sp., and *Ustilago scitaminea*	Kim et al. (2002b), Arutchelvi et al. (2008), Saravanan and Vijayakuma (2015), Morita et al. (2015)
	—		Saccharomycetes		
	—		Ustilaginomycetes		
2	Lipopeptides	—	—	—	—
2.1	—	Surfactin	Bacilli	*Bacillus subtilis, Bacillus licheniformis,* and *Bacillus mojavensis*	Arima et al. (1968), From et al. (2007), Chen et al. (2015), Pecci et al. (2010)
2.2	—	Iturin	Bacilli	*Bacillus subtilis* and *Bacillus amyloliquefaciens*	Dang et al. (2019), Romero et al. (2007)
2.3	—	Lichenysin	Bacilli	*Bacillus licheniformis*	Ali et al. (2019)
2.4	—	Viscosin	Gammaproteobacteria	*Pseudomonas fluorescens*	Alsohim et al. (2014)
2.5	—	Serrawettin	Gammaproteobacteria	*Serratia marcescens*	Sunaga et al. (2004)
2.6	—	Arthrofactin	Actinobacteria	*Arthrobacter* sp.	Morikawa et al. (1993)
2.7	—	Polymyxin	Bacilli	*Bacillus polymyxa*	Muthusamy et al. (2008)
3	Polymeric	—	—	—	—
3.1	—	Emulsan	Gammaproteobacteria	*Acinetobacter calcoaceticus RAG-1*	Zosim et al. (1982)
3.2	—	Liposan	Saccharomycetes	*Candida lipolytica*	Cirigliano and Carman (1985)
3.3	—	Biodispersan	Gammaproteobacteria	*Acinetobacter calcoaceticus A2*	Shabtai, (1990)
3.4	—	Lipomanan	Saccharomycetes	*Candida tropicalis*	Rosenberg and Ron (1999)
3.5	—	Mannoproteins	Gammaproteobacteria	*Acinetobacter* sp. and *Saccharomyces cerevisiae*	Cameron et al. (1988), Jagtap et al. (2010)
—	—	—	Saccharomycetes	—	—
3.6	—	Alasan	Gammaproteobacteria	*Acinetobacter radioresistens KA53*	Navon–Venezia et al. (1995)
4	Fatty acids, phospholipids, and neutral lipids	—	—	—	—
4.1	—	Corynomycolic acid	Actinobacteria	*Corynebacterium lepus* and *C. diphtheriae*	Fujii et al. (1999), Brennan et al. (1970)
4.2	—	Spiculisporic acid	Eurotiomycetes	*Penicillium spiculisporum* and *Talaromyces trachyspermus*	Moriwaki–takano, (2021), Ishigami et al. (1983)
	—	—	Gammaproteobacteria	—	—
4.3	—	Phosphatidyleth-anolamine	Gammaproteobacteria	*Acinetobacter* sp. and *Rhodococcus erythropolis*	Kappeli and Finnerty (1979), Kretschmer et al. (1982)
—	—	—	Actinobacteria	—	—
5	Particulate biosurfactants	—	—	—	—
5.1	—	Vesicles	Gammaproteobacteria	*Acinetobacter* sp., *P. marginilis,* and *Serratia marcescens*	Kappeli and Finnerty (1979), Matsuyama et al. (1986), Saharan et al. (2012)
5.2	—	Whole cells	Cyanophyceae	*Cyanobacteria* and many bacteria	Neufeld and Zajic (1984), Levy et al. (1990)

### Glycolipids

Glycolipids are made up of one or more carbohydrates in combination with one or more fatty acids, connected by means of ether and ester linkages. The best known glycolipids are rhamnolipids produced by *Pseudomonas* sp. (Thakur et al., 2021); trehalose lipids produced by *Rhodococcus* sp., *Nocardia* sp., *Gordonia* sp., and *Mycobacterium* sp. (Franzetti et al., 2010); and sophorolipids synthesized by *T. bombicola* and *Candida* sp. (Oliveira et al., 2015).

### Lipopeptides and Lipoproteins

These biosurfactants consist of cyclic peptides linked with the fatty acid chain. For example, surfactin, derived from *Bacillus subtilis,* consists of a seven amino-acid ring structure connected to a fatty acid chain through lactone linkage. Among other biosurfactants, it has a potent surfactant activity and has antimicrobial property against many bacteria, fungi, viruses, and mycoplasma (Meena et al., 2017). A similar biosurfactant is lichenysin produced by *Bacillus licheniformis* (Anuradha, 2010). Other biosurfactants with high surfactant and antimicrobial properties are iturin, consisting of a cyclic peptide having seven amino acid residues and 11–12 carbon containing fatty acid chain (Bonmatin et al., 2003). Viscosin is produced by *Pseudomonas fluorescens* Bak et al. (2015), serrawettin by *Serratia marcescens* Thies et al. (2014), arthrofactin by *Arthrobacter* sp. Morikawa et al. (1993), and polymyxin by *Bacillus* sp. (Banat et al., 2014b).

### Fatty Acids, Phospholipids, and Neutral Lipids

Many bacteria and yeast synthesize biosurfactants while growing on n-alkanes. For example, *Acinetobacter* sp.*,* synthesizes phosphatidylethanolamine rich vesicles which emulsify alkanes in water (Kappeli and Finnerty, 1979). Similarly, the *R. erythropolis* derived phosphatidylethanolamine reduces the interfacial tension to <1 mN/m at the hexadecane/water interface (Kretschmer et al., 1982). Spiculisporic acid, a fatty acid type biosurfactant, consists of one lactone ring and two carboxyl groups produced from *Penicillium spiculisporum* (Ishigami et al., 1983). Corynomycolic acids are biosurfactants, (R1-CH (OH)-CH (R2)-COOH) with a varied number of carbon atoms in the chain length because of the substrate in the growth medium (Fujii et al., 1999).

### Polymeric Biosurfactants

Some of the well-known polymeric biosurfactants are alasan, liposan, lipomannan, emulsan, and polysaccharide protein complexes (Saravanan and Vijayakuma, 2015). Liposan is a water-soluble molecule consisting of 83% carbohydrate and 17% protein, produced extracellularly by *Candida lipolytica* (Cirigliano and Carman, 1985)*.* Emulsan consists of three unbranched aminosugars, d-galactosamine, galactosaminouronic acid, and dideoxydiaminohexose in equal ratio with 10–22 carbon long fatty acid chain. It is mainly synthesized by *Acinetobacter calcoaceticus*, with an average molecular weight of about 1,000 kDa (Zosim et al., 1982). Alasan, a strong emulsifier produced by *A. radioresistens*, is a complex of alanine, polysaccharides, and proteins (Navon-Venezia et al., 1995). Mannoproteins are the glycoproteins, composed of proteins and carbohydrates, produced by *Acinetobacter* sp., *Saccharomyces cerevisiae*, etc. (Alcantara et al., 2013; Jagtap et al., 2010). They are strong emulsifiers and form stable emulsions with different types of oils, hydrocarbons, and have antimicrobial properties.

### Particulate Biosurfactants

This includes extracellular vesicles and whole microbial cells. Vesicles comprise of protein, phospholipids, and lipopolysaccharides. While growing on hexadecane, *Acinetobacter* sp. accumulates vesicles (20–50 nm diameter and 1.158 cubic g/cm buoyant density) on the cell surface (Kappeli and Finnerty, 1979). The whole microbial cells have both hydrocarbon and nonhydrocarbon degrading properties. For example, *A. calcoaceticus* 2CA2 acts as an emulsifier (Neufeld and Zajic, 1984).

### Genetics of Biosurfactant Production

Vast structural and functional diversity is exhibited in biosurfactants produced by microorganisms. Biosurfactant-producing strains such as *Pseudomonas, Bacillus, Acinetobacter,* and *Candida* spp. have been reported from different sources such as soil, water, and industrial effluents (Kumar and Das, 2018). Table 2 lists the various genes involved in the production of different types of biosurfactants. The genetic regulation of biosurfactant production has been mostly studied in the rhamnolipid producing strain *Pseudomonas aeruginosa*, and it is extensively shown in the literature that the biosurfactant production is induced with the involvement of quorum sensing signaling molecules (Dusane et al., 2010; Reis et al., 2011; Soberón Chávez et al., 2021).

**TABLE 2:** Genes responsible for different biosurfactant production.

S.no	Genes reported for biosurfactant	Biosurfactant type	Microorganism reported	References
1	*rhl ABRI*	Rhamnolipids	*P. aeruginosa* PG-21	Ochsner and Reiser (1995)
2	*srf A-D*	Surfactin	*Bacillus subtilis*	Cosby et al. (1995) and Menkhaus et al. (1993)
3	*licA-D*	Lichenysin	*B. licheniformis* JF2	Yakimov et al. (1995)
4	*Itu A-C*	Iturin	*B. subtilis* RB14	Tsuge et al. (2001)
5	*arf A-C*	Arthrofactin	*Pseudomonas* sp. MIS38	Roongsawang et al. (2003)
6	*gacA/gacS*	Amphisin	*Pseudomonas* sp. DSS73	Koch et al. (2002)
7	*psoA*	Putisolvin	*Pseudomonas putida* PCL1445	Dubern et al. (2005)
8	*aln A-C*	Alasan	*Acinetobacter radioresistans* KA53	Navon–Venezia et al. (1995)
9	*wza-c, wzx, wzy*	Emulsan	*Acinetobacter lwoffii* RAG-1	Nakar and Gutnick (2001)
10	*pswP*	Serrawatin	*Serratia marcescens*	Sunaga et al. (2004)
11	*emt1*	Mannosylerythritol lipids	*Candida antartica*	Kim et al. (2002a)
12	*cyp1*	Ustilagic acid	*Ustilago maydis*	Uchida et al. (1989)
13	*hfb1, hfb2*	Hydrophobins	*Trichoderma reesei*	Askolin et al. (2001)
14	*locA-D*	Locillomycin	*Bacillis subtilis* 916	Luo et al. (2015)

For biosurfactant synthesis, three enzymatic reactions occur consecutively. First, HAA (3-(3-hydroxyalkanoyloxy) alkanoic acids) is synthesized by RhlA with the esterification of acyl carrier protein (ACP)–bound two 3-hydroxyacyl molecules (produced by the fatty acid *de novo* synthesis). Second reaction is the transfer of dTDP-l-rhamnose to HAA to produce mono-rhamnolipids. The dTDP-l-rhamnose originates from glucose-6-phosphate produced by central carbon metabolism pathway. Third reaction is the production of di-rhamnolipid by joining another molecule of dTDP–l-rhamnose to the mono-rhamnolipid (catalyzed by RhlC). The expression of *rhlAB* and *rhlC* genes is regulated by Quorum sensing signaling molecules such as C_4_–HSL (*N*-(butyryl) homoserine lactone). The binding of RhlR– C_4_–HSL complex to the *rhlA* promoter activates the *rhlAB* and *rhlC* genes, which turns on the transcription of the *rhlAB* gene (Dusane et al., 2010). The following section describes quorum sensing and its link with biosurfactant biosynthesis.

## Biosurfactants and Quorum Sensing

Quorum sensing (QS) is the population density–based mechanism in which bacteria use signaling molecules for cellular communication. When the bacterial population increases, these molecules start working as autoinducers (AI). Pheromones are produced, which command their behavioral patterns and various physiological processes such as biological competence, biofilm formation, bioluminescence, antibiotic resistance, secretion of virulence factors, sporulation, and biosurfactant production (Hawver et al., 2016; Paul et al., 2018). Autoinducers (AIs) and the receptors are the two main factors required for the QS system. Autoinducers attach to the receptors which further trigger various gene regulation systems. The functioning of these QS systems involves three steps. 1) Production of signaling molecules (AIs): the microbial members produce AIs. The higher cellular density is directly proportional to the AI concentration. At lower AI concentration, signals are not recognized by the microbial system, but AI concentration above their threshold value leads to their detection and response. 2) Receptor’s accumulation: receptors present on the cell membranes, or the cytoplasmic membranes start accumulating, to increase the binding affinity with signaling molecules or for AIs detection. 3) Signal sensing: binding of AIs to receptors activates the AIs signaling systems which activate various gene expressions responsible for various factors such as virulence, pathogenicity, motility, biofilm formation, antibiotic production, biosurfactant production, etc. (Pereira et al., 2013; Seed et al., 1995).

**FIGURE 3:**
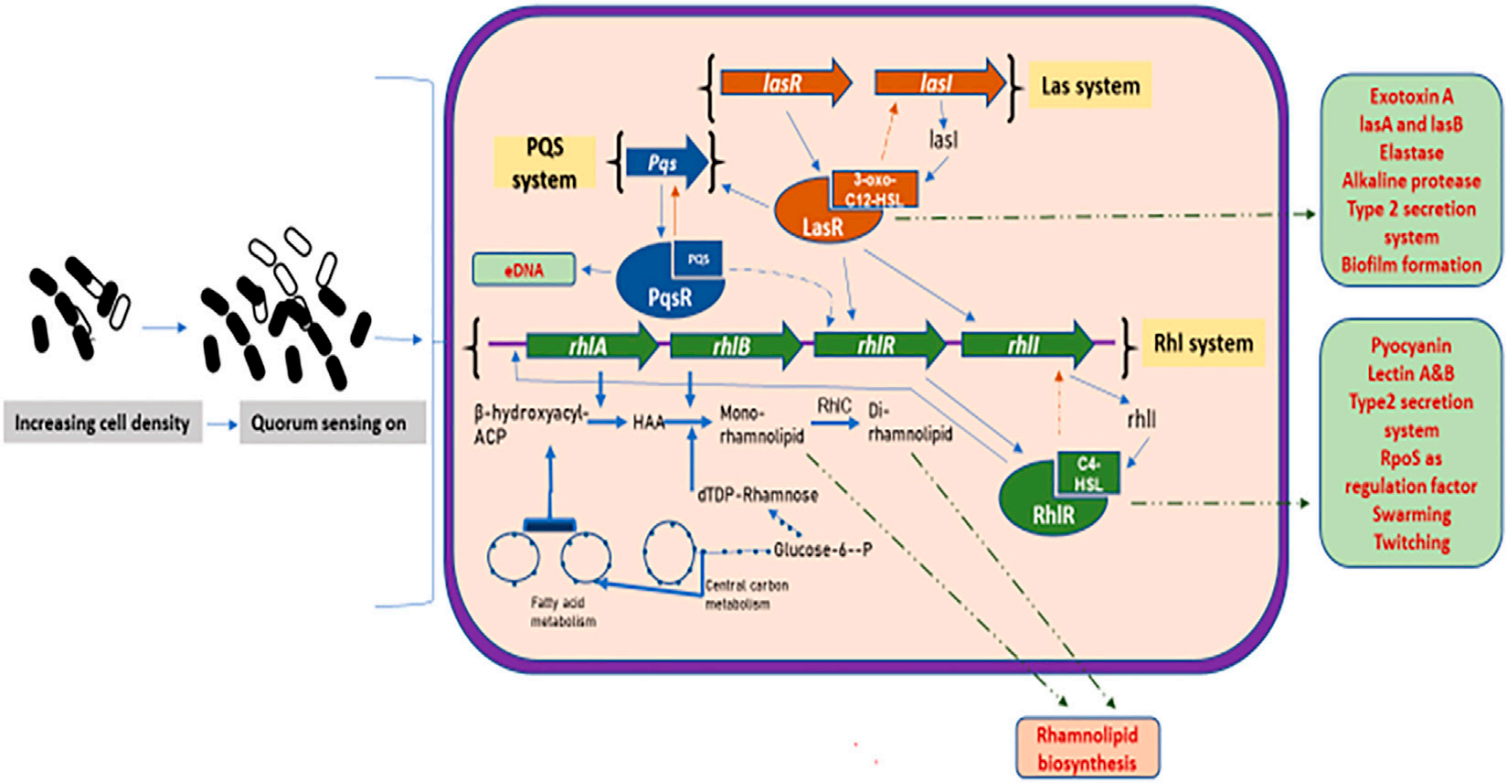
Quorum sensing system and biosurfactant biosynthesis regulation in *Pseudomonas aeruginosa*: increasing cell density activates the three major quorum sensing mechanisms, the las, rhl, and PQS. The las system has transcriptional activators, LasR and LasI (produces autoinducer 3-oxo-C_12_HSL). The 3-oxo-C_12_HSL activates LasR by complex formation leading to the induction of various genes. The 3-oxo-C_12_HSL–LasR complex also activates PQS system, where PQS binds to its receptor, PqsR, thus regulating the expression of various virulence factors and biofilm formation. The rhl system has rhlA and rhlB that produces rhamnosyltransferase, arranged with transcriptional activators, rhlR and rhlI (produce autoinducer C_4_–HSL). The autoinducer C_4_–HSL acts and forms complex with RhlR, which induces gene expression of various factors. The C_4_–HSL–RhlR complex also activates the rhlA promoter, leading to the transcription of the rhlAB genes to produce rhamnolipids. Thick blue lines depict the sequential reactions for the formation of mono- and di-rhamnolipids, also showing the biosynthesis routes to produce their dTDP–rhamnose and fatty acid precursors. Green dashed lines depict the transport of various gene factors outside the cell membrane.

It has been shown that in *P. aeruginosa,* two pair of genes known as *LasI/LasR* and *RhlI/RhlR* function in series to control the expression of various factors such as virulence, biofilm formation, antibiotic production, biosurfactant production, and motility. The *P. aeruginosa* has interlinked las, rhl, and PQS systems (Sullivan, 1998; Dusane et al., 2010). The rhl system catalyzes the synthesis of an HSL autoinducer, *N*-(butyryl) homoserine lactone (C4-HSL), which is regulated by the RhlR and RhlI (transcriptional regulators). Similarly, the las system catalyzes the formation of another HSL autoinducer, N-(3-oxododecanoyl)-homoserine lactone (3-oxo-C_12_–HSL). As the cell density increases, the signaling molecule, 3-oxo-C_12_–HSL, accumulates, binds to the LasR protein, and forms LasR-3-oxo-C_12_-HSL complex. This complex binds to the promoter sequences of the preceding genes which encodes for various virulence factors such as elastase, protease, exotoxinA, and alkaline phosphatase. This complex also activates the expression of rhlR*,* which in turn activates the second quorum sensing system called the rhl system. The RhlR binds to the RhlI-directed autoinducer called C_4_–HSL to form an RhlR– C_4_–HSL complex which induces the expression of genes responsible for functions such as pyocyanin antibiotic synthesis, cytotoxic lectin synthesis, motility, and biosurfactant synthesis (Maier and Soberón–Chávez, 2000). The PQS system acts as a regulatory link between the rhl and las systems (Soberón–Chávez et al., 2021). The PQS system requires LasR for its gene expression, inducing the expression of the rhlI, which is responsible for the production of autoinducer C_4_-HSL. Figure 3 shows a model showing the quorum sensing system and biosurfactant biosynthesis in *P. aeruginosa*.

Besides *N*-(butryl) homoserine lactone, QS systems use various other signaling molecules for communication. For example, for intraspecies communication in case of Gram-negative bacteria, N-acyl-homoserine lactones, 3-hydroxypalmitic acid methyl ester, and *cis*-unsaturated fatty acids are used. Similarly, gamma-butyrolactones, and cyclic peptides are used for communication in case of Gram-positive bacteria. For interspecies communication, C4-HSL, N-butanoylhomoserine lactone, 3-oxo-C12-HSL, peptide lactones, and peptide thiolactones signaling molecules are used (Ng and Bassler, 2009; Quadriya et al., 2018).

Microorganisms use QS-mediated biosurfactant production for various activities such as recovering impaired motility, virulence, biofilm formation, or deformation. The quorum sensing system controls the biosurfactant production (Kumar and Das, 2018). However, biosurfactant controls the quorum sensing signaling (intercellular and intracellular communication) and quorum sensing–dependent activities such as biofilm formation, motility, and pathogenicity (Abisado et al., 2018; Mangwani et al., 2016). For example, in *Pseudomonas putida*, AHL-producing QS system is regulated by ppuI, rsaL, and ppuR*,* which are involved in biofilm formation and production of lipopeptides. Mutation in ppuI and ppuR increases biofilm formation and an rsaL mutant overproduces lipopeptides and controls the biofilm formation (Dubern et al., 2006; Steidle et al., 2002). A cyclic lipopeptide, amphisin’s production is also regulated by QS in *Pseudomonas* sp. DSS73. The biosurfactant has antifungal properties against *Pythium ultimum* and *R. solani* (Andersen et al., 2003). A biosurfactant produced by *Cobetia* sp. interferes with the lipophilic signals involved in intercellular communication, by causing the repression of virulence genes (*aero and sat A*) and genes for biofilm formation in its potential competitor *A. salmonicida* (a fish pathogen). Both of these genes rely on quorum sensing for their functioning (Ibacache–Quiroga et al., 2013). Thus, biosurfactants interfere in quorum sensing signaling and affect the intra/intercellular communication. Some studies have shown the impact of biosurfactant synthesis efficiency on the cellular communication mediated by QS (high cell density), establishing the connection between high cellular population density and biosurfactant production (De Dier et al., 2015). The increased concentration of autoinducer molecules correlates with the increasing concentration of rhamnolipids produced by the bacterial cultures isolated from feces (Woźniak-Karczewska et al., 2017). Impacts on the rhamnolipid concentration by different mutations in QS systems of *Burkholderia thailandensis* was observed with different growth phases and conditions (Victor et al., 2019). High biosurfactant production is observed with the high concentration of autoinducer molecules, which increased cellular communication and growth of the microbial cells in the system (Nakata et al., 1998).

## Biosurfactants and Motility

Surface-associated bacteria are highly motile and migrate at a higher rate over the substrate. This process is known as swarming. Swarming motility is the rapid movement of bacteria across a surface or energy-rich solid medium fueled by rotating flagella. It is reported that many swarming bacteria produce surfactants which help in reducing the surface tension between the surface and the bacterial cells, which allows them to spread over the surfaces (Verstraeten et al., 2008). Such a cellular behavior is controlled by the QS system. These behaviors include functional connections between swarming motility driven by flagellum and surfactant production, chemotaxis behavior, virulence, and biofilm production. Many studies have shown that biosurfactant production and the flagellar biosynthesis play an important role in swarming motility along with the cellular communications (Kearns, 2010). For example, in *B. subtilis*, a mutation in the flagellar biosynthesis gene or in the surfactin production gene (*srfAA*), resulted in the loss of swarming motility in the mutants (Julkowska et al., 2005; Kearns and Losick, 2003). Similarly, *Serratia* spp. produces serrawettin (lipopeptide type surfactant), and mutations in the genes that are responsible for its biosynthesis led to the abolishment of swarming motility (Lindum et al., 1998). *P. aeruginosa* produces rhamnolipids which are divided into HAA (β-hydroxydecanoyl-β-hydroxydecanoate) and mono or di-rhamnolipids which act as a surfactant to promote swarming (Wang et al., 2014). Apart from these, there are some Gram-negative bacteria which do not require surfactants for swarming. For example, *E. coli*, *S. enterica*, *and P. mirabilis* can swarm by using the LPS (lipopolysaccharide) present in their outer membrane as their wetting agent. It has been shown in various studies that LPS deficient mutants are unable to swarm (Harshey and Matsuyama, 1994).

### Mechanism of Regulating Bacterial Motility by Biosurfactants

*Pseudomonas aeruginosa* exhibits three types of motility: twitching, swimming, and swarming (Harshey, 2003). For the colonial movement on a semisolid surface, it needs functional flagellar movement and biosurfactant production (Verstraeten et al., 2008). Genetic analysis revealed that both these processes are inter-linked (Kearns, 2010). As mentioned above, *P. aeruginosa* produces biosurfactants, consisting of HAA and mono- or di-rhamnose sugar moieties. RhlA enzyme is responsible for the synthesis of the HAA part, and RhlB and RhlC convert it into mono- and di-rhamnolipids, respectively. It is very well established in the studies that *rhlAB* operon and HAA synthesis are interlinked with flagellar biosynthesis genes, and hence affect cellular motility (Kohler et al., 2000; Déziel et al., 2003). Any mutations in the flagellar biosynthesis genes can directly affect HAA production. For example, any disruption in the Class 1 flagellar gene, which includes *fleQ* (transcriptional factor and important flagellar gene regulator) and fliA (codes for the sigma factor σ^28^), results in the complete loss of HAA production. The *fleQ* gene transcriptionally activates the *rhlA* gene, which leads to the production of HAA. Similarly, fliA also increases the production of HAA (Burch et al., 2012).

The disruption of Class 2 genes which includes *fleSR* (response regulators, activated by *fleQ* transcriptional response) and *fliF* (genes encoding for MS ring), and Class 3 genes (*flgC, flgD*) results in a reduced surfactant production (Burch et al., 2012). Any disruption in Class 4 genes which includes *fliC* (flagellin) and genes for chemotaxis causes the overproduction of biosurfactants (Xu et al., 2012). An increase in expression of Class 4 genes or flagellin (*fliC*) results in a decreased production of HAA.

**FIGURE 4:**
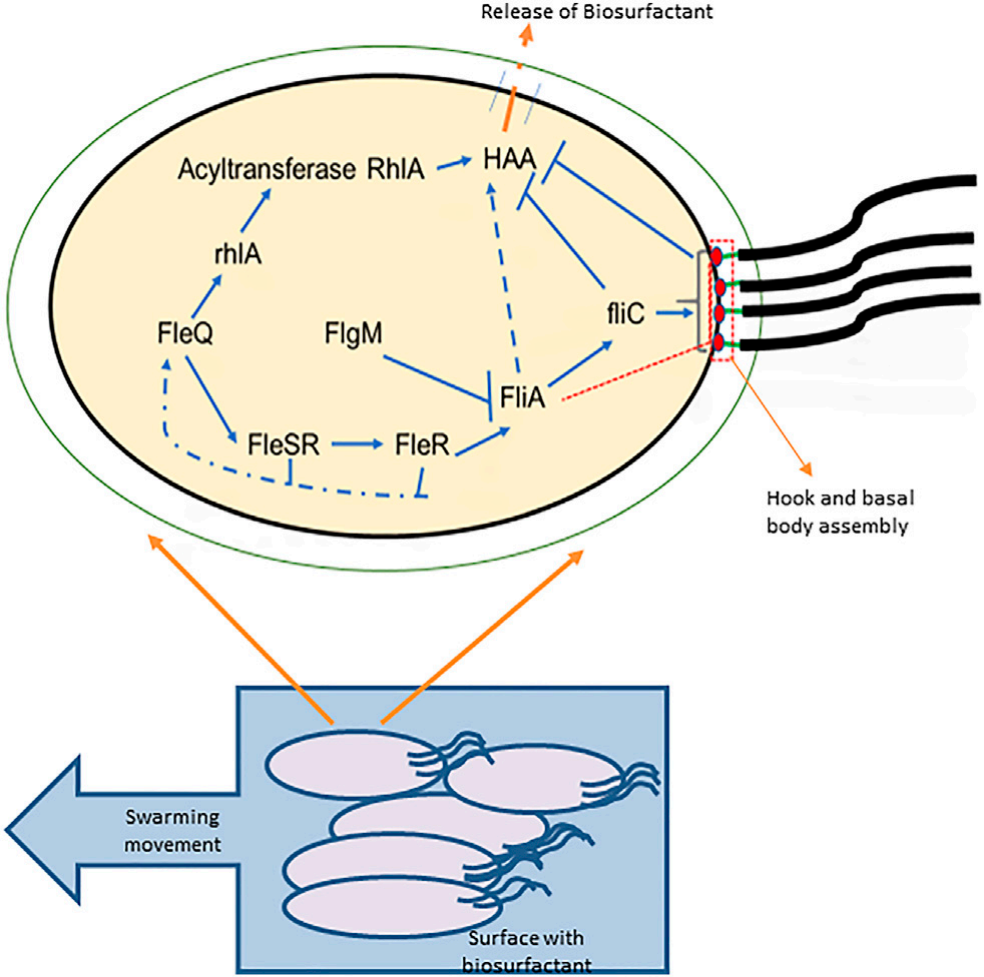
Correlation between motility and HAA production: a bacterial cell is shown with the flagella required for motility and with the four-tiered flagellar regulatory hierarchy and HAA production. FleQ transcriptionally activates FleSR, which is required for site selection and formation of hook and basal body complex formation. After the complete formation of hook and basal body complex, an anti-sigma factor is secreted through it (indicated in red dashed box and line), releases the FliA inhibition which directs the genetic expression of FliC (flagellin) and chemotaxis proteins, and controls the flagellar motor rotation. FleQ also controls the HAA formation from the acylated precursors by the transcriptional activity of rhlA. HAA is transported outside through cell membrane (shown with orange dashed line). Flagella synthesis depicts that the early flagellar assembly activates HAA production, but the late stages including fliC activation and flagellar maturation restrict the HAA production (Adapted from (Xu et al., 2012)).

When a surfactant is enough in the external environment for reducing the surface tension, it is sensed by the flagella, and cell locomotion is promoted. This shows that flagellar assembly can incite the HAA production (Figure 4). This mechanism is proven by various studies involving the disruption of the various genes (Wang et al., 2014). For example, in *P. aeruginosa* and *P. syringae* mutation in any of the flagellar genes affects the HAA production (Burch et al., 2012).

## Biosurfactant and Virulence

Many microorganisms have the capability to adapt to a wide range of habitats because of their ability to produce various virulence factors such as pyocyanin, elastases, proteases, etc. Virulence factors are responsible for pathogenicity as they facilitate adhesion or dispersion of biofilms to host cells. It is established that the production of various virulence factors is related to the biosurfactant producing genes (Pearson et al., 1997; Maier and Soberón–Chávez, 2000). Most of the biosurfactant-producing organisms are pathogenic, for example, rhamnolipid-producing *P. aeruginosa, Burkholderia cepacian,* and *Burkholderia pseudomallei*; phospholipid-producing *Klebsiella pneumoniae*; lipopeptide-producing *Serratia marcescens*; heteropolysaccharide-producing *Cronobacter sakazakii*; and glycolipid-producing *Nocardia otitidiscaviarum, Alcaligenes faecalis*, etc. (Uzoigwe et al., 2015).

*Pseudomonas aeruginosa* is responsible for various infections in immune-compromised individuals. Production of disease-causing virulence factors is regulated in correlation with biosurfactant biosynthesis and regulatory system (las/rhl system). As described previously, at high cell concentration, AI 3OC_12_HSL (3-oxo-C_12_-homoserine lactone) binds to LasR, which in turn activates the production of various virulence factors by binding to the promotors of various preceding genes such as *lasB* coding for elastase, *lasA* coding for protease, *toxA* coding for exotoxinA, and *aprA* coding for alkaline phosphatase and *rhlI*, which produces another AI called C4-HSL (Lee and Zhang, 2015). The AI C4-HSL binds to RhlR, which also activates the production of various virulence factors such as *lasB, aprA,* and *rpoS* coding for the sigma factor involved in the synthesis of rhamnolipid, *lecA* coding for cytotoxic lectin, and genes for pyocyanin (Strateva and Mitov, 2011; Moradali et al., 2017).

The major virulence factor regulator, VqsR protein, acts as a homolog to LuxR. In VqsR mutants, a decrease in rhamnolipid synthesis was observed (Juhas et al., 2004). Another virulence factor regulator is Vfr, any mutation in Vfr affects the LasR/RhlR production, therefore, affecting the expression of multiple virulence factors such as protease, exotoxin and rhamnolipid production (Reis et al., 2011). PtxR is a transcriptional regulator of exotoxin A gene toxA*.* Mutation in ptxR increases the production of pyocyanin. It also regulates the rhamnolipid production by repressing the expression of the PQS genes. In ptxR mutants enhanced rhlI and reduced lasI gene expression was observed. Due to higher rhlI expression, level of C_4_–HSL concentration increases, which further lead to the activation of RhlR (RhlR–C_4_–HSL). This results in the enhanced transcription expression of rhlA, further leading to enhanced rhamnolipid production (Carty et al., 2006). Virulence factors like alginate and lipopolysaccharide (LPS) production are associated with an essential protein, AlgC, required for the synthesis of rhamnolipids (Olvera et al., 1999). Another study demonstrated the effect of rhamnolipids on mature biofilms resulting in their disassembly and significant increase in the production of virulence factors such as protease and siderophore in *Burkholderia pseudomallei* (Sidrim et al., 2020)*.*

## Biosurfactant Role in Antagonism and Their Mechanism of Action

Biosurfactants can induce pore and ion channel formation in a lipid bilayer membrane, which destabilizes or disturbs the integrity and permeability of the membranes. This results in the disruption of membrane and cell death. This mode of action results in the biological activities of biosurfactants which include, antibacterial, antifungal, antiviral, and antimycoplasma (Fracchia et al., 2012). This biological activity depends on their different structures (Kracht et al., 1999).

Lipopeptide (LP) type of biosurfactants, such as surfactin, fengycin, polymyxins, etc., possesses antimicrobial activities. It is reported that lipopeptides of lipid tail length of 10–12 carbon atoms have an antibacterial activity and lipid tail length of 14–16 carbon atoms increases the antifungal activity (Malina and Shai, 2005). Lipopeptides have the property to form micellular aggregates or pore channels in the lipid membrane, causing membrane disruption, increased membrane permeability, increased metabolites leakage, membrane structure change, change in protein conformations, altering membrane functions, cell lysis, and cell death. Dimerization of surfactin into the membrane bilayer causes cellular membrane leakage and destabilization. When tested *in vitro,* surfactin was found to be incorporated into the membrane, causing the dehydration of the head groups of the phospholipid resulting in distorted or altered membrane barrier properties of the lipid bilayer. Surfactin confers the competitive advantage in interactions with different viruses. It exposes the capsid of the virus particles by acting on the viral envelope, leading to the leakage and disintegration of the viral envelope (Carrillo et al., 2003). Polymyxins display antimicrobial activity against Gram-negative bacteria by binding to the lipid A component of lipopolysaccharide to increase the permeability and disruption of the plasma membrane (Velkov et al., 2010). Another lipopeptide, iturin, also exhibits antibacterial activity like surfactin and antifungal property as well. It is mediated by the interaction between the surfactant molecule and sterol components present in the membrane, which causes *trans*-membrane permeability (Zohora et al., 2013; Hiradate et al., 2002). Other lipopeptides such as fengycin, mycosubtilins, viscosin, etc., also exhibit a similar type of activity with other microorganisms (Deleu et al., 2008; Romero et al., 2007). LPs produced by *Bacillus* sp. also show antibacterial property toward many Gram-negative and Gram-positive bacteria like *B. megaterium, Mycobacterium tuberculosis, Mycobacterium smegmatis, B. cereus, P. syringae,* etc., and antifungal activity toward *Aspergillus flavus, Colletotrichum gloeosporioides, Fusarium verticillioides, R. solani, Fusarium graminearum, Penicillium roqueforti,* etc. LPs have activity against many algae such as *Pythium, Phytophthora infestans, Phytophthora capsica,* etc. (Raaijmakers et al., 2010).

Glycolipids also exhibit competitive advantages in connection with many other microorganisms. Rhamnolipids cause reduction in LPS content in membranes, increase cell hydrophobicity, cause changes in membrane proteins, and disturb surface morphology. A study revealed that di-rhamnolipid structures have a large polar head and a small hydrophobic part, which acts as an inverted-cone shaped molecule on the cell membrane by extending a positive curvature on it and disrupts the membrane (Ortiz et al., 2010). Another study demonstrated that di-rhamnolipids interact with the phospholipid part of the membrane, which resulted in an alteration in the acyl chain and disturbed the integrity of the bilayer membrane (Sánchez et al., 2009). Similarly, other glycolipids such as sophorolipids and trehalose lipids have a similar mechanism which leads to the destabilization, change in permeability, loss of membrane functions, and structural changes (Zaragoza et al., 2009; Joshi-Navare and Prabhune, 2013). Some of the examples of glycolipids with antibacterial activity include, rhamnolipids against *Staphylococcus aureus, Proteus vulgaris, Streptococcus faecalis, Serratia marcescens, Enterobacter aerogenes, Klebsiella pneumoniae, Micrococcus Luteus,* etc. (de Araujo et al., 2016; Thakur et al., 2021). Sophorolipids have antibacterial activity against *B. subtilis, Bacillus circulans, Streptococcus agalactiae*, *Rhodococcus erythropolis,* etc. (Joshi-Navare and Prabhune, 2013; Sleiman et al., 2009; K. Kim et al., 2002a). Glycolipids act as antifungal agents against *Phytophthora capsica, Phytophthora cryptogea, Botrytis cinerea, Saccharomyces* sp. *Pencillium, Aspergillus, Mucor* spp., etc. (Inès and Dhouha, 2015). Glycolipids function as an antiviral agent by suppressing their growth against herpesvirus, tobacco mosaic virus, and by showing immunomodulatory effects against influenza virus, etc. (Haferburg et al., 1987; Remichkova et al., 2008; Fracchia et al., 2012).

## Biosurfactant Role in Biofilm Formation and Removal

The structural aggregation of the bacterial cells by adhesion over the different surfaces is known as biofilm (Trafny, 2008). Different processes are responsible for the formation of the biofilm (Kirov, 2003). The first step is the formation of the conditioned film which helps in the attachment of microbial cells. The release of biosurfactants can promote the film conditioning on abiotic and biotic surfaces by altering their physical and chemical nature. The second step is the formation of the microcolony by transporting the microbes and nutrients to the surface. Bacterial motility, Brownian motion, and molecular diffusion help in adhesion of bacteria (Kirov, 2003). The third step is the maturation of the biofilm due to the release of various factors such as the stabilization of the matrix by extracellular polymeric matrix (EPS), cell surface hydrophobicity to increase the rate of adhesion, and motility. In *P. aeruginosa*, release of all these factors is regulated by the Las/Rhl/PQS QS circuit system. The presence of rhamnolipids increases the cell surface hydrophobicity. Development and multiplication of microbes over the host surface are linked with the EPS production. After development, cellular communication between the biofilm-forming cells starts, which leads to the production of various virulence factors and pathogenesis. Biofilms are advantageous in some applications such as bioremediation and waste-water treatment. Biosurfactants help in conditioning of the film and modification of the bacterial surface hydrophobicity, and therefore help in bacterial adhesion to surfaces for biofilm formation (Quadriya et al., 2018). For example, a study demonstrated the influence of lipopeptide biosynthesis on the decreasing cell surface hydrophobicity and on the adhesion of *Bacillus* spp. to stainless steel (Czaczyk et al., 2008). Another study showed the essential role of biosurfactants in bacterial adsorption and biofilm formation at the ƞ-decane–water interface by *P. aeruginosa, S. aureus,* and *S. epidermidis* (Subbiahdoss and Reimhult, 2020). Biosurfactants also help in the detachment of microbial cells from the surfaces by erosion, sloughing, and abrasion (Quadriya et al., 2018). Because of this property, they can be used as a strong anti-adhesive agent to prevent microbial infections (Brindhadevi et al., 2020). Biosurfactants act as anti-biofilm agent by showing its inhibitory effects. Such an anti-biofilm activity of rhamnolipids, sophorolipids, and lipopeptides has been observed against dual species (fungal/bacterial) such as *Candida albicans, S. aureus,* and *S. epidermidis* (Ceresa et al., 2021). They form dense biofilms, which are responsible for chronic infections. The lipopeptide type biosurfactant produced by *Bacillus tequilensis* SDS21 exhibit anti-biofilm activity by dislodging biofilm from stainless steel, glass, and polystyrene surface(A. K. Singh and Sharma, 2020). In a recent report, many biosurfactants isolated from different microorganisms and their action as an anti-biofilm agent have been described (Mishra et al., 2020). Thus, they play a role in cellular communication by exhibiting inhibitory activity against unwanted species (Mishra et al., 2020). This property makes biosurfactants useful as a substitute for chemical surfactants in various industries such as cosmetics, medicine, etc. (Adu et al., 2020; da Silva et al., 2021).

### Conclusion and Future Prospects

Biosurfactants have multiple roles in microbial physiology and environmental processes such as motility, quorum sensing, intercellular antagonism, pathogenesis, biofilm formation and its maintenance, intracellular bioavailability and efflux of nutrients, toxic compounds, regulatory molecules, and gene regulation. In this article, we have discussed about the different types of biosurfactants and their natural roles in cellular community and communication behavior. To date, many structural and regulatory genes have been identified for the production of various biosurfactants. Among all, the genetic regulation of only rhamnolipid and surfactin has been well studied. A better understanding of genetic regulation of other types of biosurfactants is required to establish their role with other microbial functions. Microorganisms use communication mechanism based on cellular density, known as QS systems, to control various specific cellular functions. The QS system and its involvement in other cellular mechanisms in various microbial systems, especially in the production of biosurfactants could be used in the accomplishment of its industrial scale production. The biosurfactant-producing genes are also linked with cellular locomotion, biofilm formation, pathogenesis, etc., in various bacteria which help in establishing inter- or intracellular communication. Our current understanding of the scope and importance of cellular communication mechanisms and their gene regulation to produce biosurfactants is still in its infancy stage. Now, the biosurfactant market is growing and expanding its usage in various applications, but the biosurfactants produced from the microorganisms are low in quantity and the downstream processing costs around 70% of total expenditure. So, there is a need to establish the mechanisms of genetic regulation, study the biochemistry of biosurfactant biosynthesis enzymes, and understand their role in cellular communication in microbial community. This could result in a common environmental cue to trigger the biosurfactant production and to reduce the production cost.
